# How Sublaminar Bands Affect Postoperative Sagittal Alignment in AIS Patients with Preoperative Hypokyphosis? Results of a Series of 34 Patients with 2-Year Follow-Up

**DOI:** 10.1155/2016/1954712

**Published:** 2016-11-23

**Authors:** Sébastien Pesenti, Antoine Chalopin, Emilie Peltier, Elie Choufani, Matthieu Ollivier, Stéphane Fuentes, Benjamin Blondel, Jean-Luc Jouve

**Affiliations:** ^1^Pediatric Orthopedics, Timone, Aix-Marseille University, 264 rue St Pierre, 13005 Marseille, France; ^2^Aix-Marseille University, CNRS, ISM, Inst Movement Sci, Marseille, France; ^3^Spine Unit, Timone, Aix-Marseille University, 264 rue St Pierre, 13005 Marseille, France

## Abstract

Hypokyphosis is currently observed in thoracic idiopathic scoliosis. The use of sublaminar bands allows a good restoration of sagittal balance of the spine. The aim of the study was to provide a middle-term radiographic analysis of patients with adolescent idiopathic scoliosis with preoperative hypokyphosis treated by posterior arthrodesis with sublaminar bands. This retrospective study included 34 patients with Lenke 1 scoliosis associated with hypokyphosis (TK < 20°). A radiographic evaluation was performed with a 2-year follow-up. Cobb angle, cervical lordosis, thoracic kyphosis, lumbar lordosis, and pelvic parameters were measured preoperatively, postoperatively, and at 6-month and 2-year follow-up. The mean preoperative thoracic kyphosis was 10.5° versus 24.1° postoperatively (*p* < 0.001), representing a mean gain of 13°. Cobb angle ranged from 59.3° to 17.9° postoperatively (mean correction 69%, *p* < 0.001). Cobb angle increased between the immediate postoperative measurement and the 6-month follow-up (17.9 versus 19.9, *p* = 0.03). Cervical curvature changed from a 5.6° kyphosis to a 3.5° lordosis (*p* = 0.001). Concerning lumbar lordosis, preoperative measurement was 39.7° versus 41.3° postoperatively (*p* = 0.27). At 6-month follow-up, lumbar lordosis significantly increased to 43.6° (*p* = 0.03). All parameters were stable at final follow-up. Correction performed by sublaminar bands is efficient for both fontal and sagittal planes. Moreover, the restoration of normal thoracic kyphosis is followed by an adaptation of the adjacent curvatures with improved cervical lordosis and lumbar lordosis.

## 1. Introduction

The 3-dimension deformity of the spine that occurs in adolescent idiopathic scoliosis often induces a decrease of sagittal curvatures. In particular in thoracic curves, hypokyphosis is currently observed [1–3]. In adults, it has been widely demonstrated that improvement of sagittal curvatures of the spine and restoration of a correct sagittal alignment were significantly correlated to better surgical outcomes and quality of life. In younger patients, sagittal balance seems to have also a great importance for the future quality of life.

Thus, the aim of surgery in adolescent idiopathic scoliosis is to restore both frontal and sagittal alignment. Since Cotrel-Dubousset technique has been described, a lot of instrumentation has been used to reach this goal. But the kind of device to use remains controversial. In 2009, Mazda et al. have introduced the use of a new device: the sublaminar bands [4]. The principle of reduction with these bands is the posteromedial translation of the spine. Recent literature suggests that the use of this kind of hybrid instrumentation allows a good correction of the curvature in the frontal plane but also a better restoration of sagittal balance of the spine [5–7]. The hypothesis of this work was that hybrid instrumentation using sublaminar bands was efficient in the restoration of sagittal alignment in patients with adolescent idiopathic scoliosis and with hypokyphosis.

The aim of the study was to provide a short- to middle-term radiographic analysis of patients with adolescent idiopathic scoliosis with preoperative hypokyphosis treated by posterior arthrodesis with sublaminar bands.

## 2. Method

### 2.1. Population

It was a retrospective study. Inclusion criteria were (1) adolescent idiopathic scoliosis Lenke 1 or 2, (2) preoperative kyphosis T4–T12 < 20°, (3) posterior arthrodesis using hybrid construct with sublaminar bands, and (4) minimum follow-up of 2 years. Patients with other kinds of scoliosis, with a thoracic kyphosis > 20°, operated on with anterior or combined approach, or with other constructs were excluded from the study.

From January 2011 to December 2013, 194 patients with adolescent idiopathic scoliosis have been operated on in the pediatric orthopedic department of the institution. Among these 194 patients, 34 met the inclusion criteria.

### 2.2. Surgical Technique

**Figure 1:**
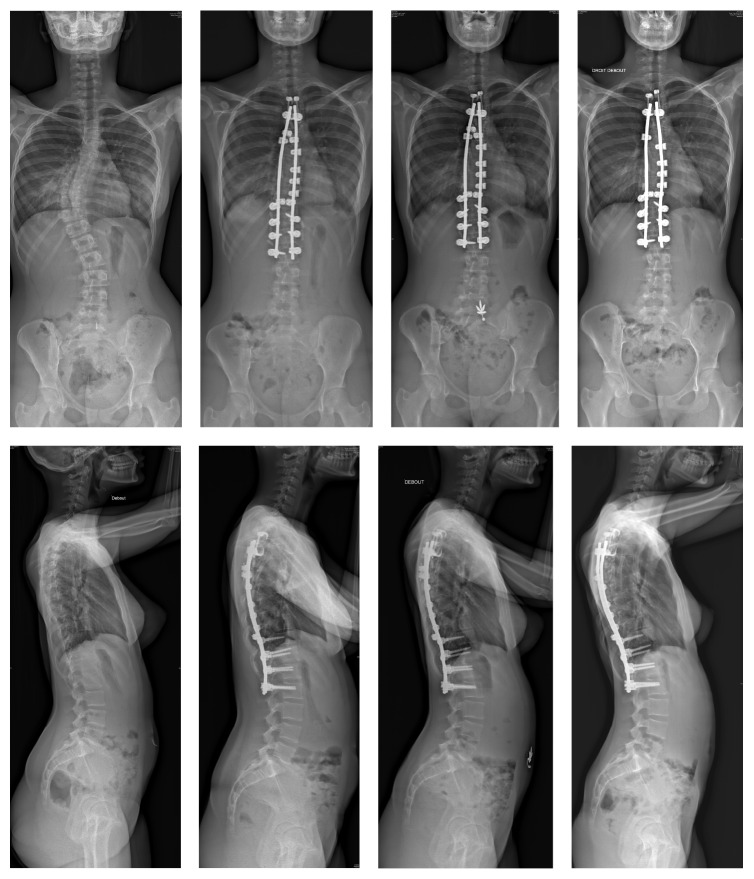
Preoperative, postoperative, and 2-year follow-up radiographs. The patient presented a Lenke 1 scoliosis with severe hypokyphosis. Hybrid instrumentation with sublaminar bands allowed correction of the deformity in frontal plane but also the restoration of the thoracic kyphosis. At last follow-up, the correction in both frontal and sagittal planes is maintained and sagittal alignment is satisfactory.

Arthrodesis was performed by posterior approach only, in prone position. Two laminolaminar claws were placed on the 2 upper instrumented vertebras. Bilateral pedicular screws were placed at the distal end of the construct. Two titanium rods were then connected to the claws and to the pedicular screws to obtain a rigid frame. Thoracic vertebras were instrumented with one sublaminar band per level, attached to the rod in the concavity. One extra sublaminar band was placed at the apex of the kyphosis and attached to the rod in the convexity to increase the posterior translation effect. The tensioning of the bands allowed the reduction of the curvature in both frontal and sagittal plane by posteromedial translation maneuvers (Figure 1).

### 2.3. Radiographic Evaluation

Radiographic evaluation was performed preoperatively, postoperatively, and with 6-month and 2-year follow-up. Measurements were made from low-dose stand-up AP and lateral radiographs (EOS, Paris, France). Measurements were made by 3 independent observers.

On AP radiographs, the main Cobb angle was measured. On lateral radiographs, the following parameters were measured:

(i) Cervical lordosis: angle is between the lower endplate of C2 and the lower endplate of C7.
(ii) Thoracic kyphosis: angle is between the upper endplate of T4 and the lower endplate of T12.
(iii) Lumbar lordosis: angle is between the upper endplate of L1 and the lower endplate of L5.
(iv) Pelvic incidence, pelvic version, and sacral slope: pelvic incidence (PI) was considered as correctly correlated to the lumbar lordosis (LL) if the ratio LL = PI ± 10 was respected.
(v) SVA: distance is between C7 plumb line and the posterosuperior edge of S1 endplate.

### 2.4. Statistical Analysis

Data were formulated as means, ranges, and standard deviations. Comparison of means used the Student *t*-test for normally distributed variables, or otherwise nonparametric Wilcoxon test. For qualitative data, the Chi2 test was used. For correlations, a Pearson correlation test was used. The significance threshold was set at *p* < 0.05.

## 3. Results

A total of 34 patients were included in the study. There were 28 girls and 6 boys. Mean age at intervention was 15.4 years old ± 1.9 years (from 12.4 to 21 y.o.). The mean number of level fused was 12 ± 1 (from 11 to 15).

The results of radiographic measurements are summarized in Table 1.

### 3.1. Frontal Plane

The mean preoperative Cobb angle was 59.3° and 17.9° postoperatively. The mean correction rate was 69%. The difference between preoperative and postoperative Cobb angle was significant (59.3° versus 17.9°, *p* < 0.001). At 6-month follow-up, the mean Cobb angle was 19.9° and 20.1° at 2-year follow-up. The difference between immediate postoperative and 6-month follow-up Cobb angles was significant (17.9° versus 19.9°, *p* = 0.003).

### 3.2. Sagittal Curvatures

The mean preoperative thoracic kyphosis was 10.5° and 24.1° postoperatively. The mean gain of thoracic kyphosis was 13° ± 8.4 (from 2 to 41°). The difference between preoperative and immediate postoperative thoracic kyphosis was significant (10.5 versus 24.1, *p* < 0.001). The measurements at 6-month and 2-year follow-up did not show significant differences.

The mean cervical curvature ranged from a 5.6° kyphosis preoperatively to a 3.5° lordosis postoperatively. This difference was significant (−5.6° versus 3.5°, *p* = 0.001). This parameter was stable at 6-month follow-up. At 2 years, there was a significant increase of cervical lordosis, along with the change of lumbar lordosis (4.3 versus 9.4, *p* = 0.02).

Concerning lumbar lordosis, there was no significant difference between pre- and postoperative measurement. At 6-month follow-up, lumbar lordosis was 43.6°. The difference between immediate postoperative and 6-month follow-up lordosis was significant (41.3° versus 43.6°, *p* = 0.03). At 2-year follow-up, lumbar lordosis was stable.

The changes in lumbar lordosis between the pre- and the postoperative measurements were not significantly correlated (*R* = 0.286, *p* = 0,107). However, this correlation was significant at 6 months and 2 years (resp., *R* = 0.378, *p* = 0,04 and *R* = 0.36, *p* = 0,04).

### 3.3. Pelvic Parameters

The mean preoperative pelvic incidence was 45.2° ±  11.8 and did not change throughout follow-up.

Concerning pelvic version, there was a significant increase between pre- and immediate postoperative measurements (5.5 versus 8.5, *p* = 0.02). These changes were transitory and the difference between preoperative and 2-year follow-up measurements was not significant (5.5 versus 7, *p* = 0.13).

The mean preoperative PI-LL was 4.2 ± 13. Preoperatively, 14 patients had a lumbar lordosis not correctly correlated to their lumbar lordosis (PI-LL > 10). The mean PI-LL at 2-year follow-up was 0.3 ± 7.3. At final follow-up, 4 patients had a lumbar lordosis not correctly correlated to their pelvic incidence. The number of patients with a lumbar lordosis in accordance with the pelvic incidence was significantly higher at 2-year follow-up (*p* = 0.009).

### 3.4. Global Sagittal Alignment

Preoperative SVA was 1.3 cm ± 2.3. Postoperatively, mean SVA was 1.4 cm ± 2.9. This difference was not significant (1.3 versus 1.4, *p* = 0.72). At 6-month follow-up, mean SVA was 0.9 cm ± 1.7. At 2-year follow-up, mean SVA was 0.3 cm ± 1.7. When compared to preoperative measurements, this value was significantly lower (1.3 versus 0.3, *p* = 0.02).

## 4. Discussion

### 4.1. Correction of Frontal Plane

Efficiency of sublaminar bands for the correction of scoliotic deformities in adolescents has been demonstrated by many authors, even in patients with preoperative hypokyphosis [4, 5, 8–10]. In this series, the mean correction rate of the Cobb angle was almost 70%. This result is similar to those reported in the literature using sublaminar bands. Furthermore, the efficiency of sublaminar bands for the correction of the frontal plane is comparable to the other kind of construct, including all-pedicular screw constructs [7].

In this series, it is interesting to note that the Cobb angle increased significantly between immediate postoperative and 6-month follow-up. The correction was stable between 6-month and 2-year follow-up. The relative flexibility of this kind of construct may explain the loss of correction during the first months, while the fusion is not yet acquired. However, the mean loss of correction during the first 6 months was only 2°, which is not clinically relevant. This finding is also observed with the all-screw constructs, with an average loss of correction of 4% during the 2 first years [11].

### 4.2. Correction of Thoracic Kyphosis

The use of sublaminar bands has permitted a significant increase of postoperative thoracic kyphosis. These results are consistent with those described in the literature [4, 5, 8–10] and confirm the efficiency of posteromedial translation maneuvers for the restoration of thoracic kyphosis in hypokyphotic patients. Fletcher et al. have demonstrated that all-screw constructs were less efficient than hybrid constructs in the restoration of thoracic kyphosis [12]. Furthermore, some authors have introduced the fact that the vertebral derotation induced by pedicular screws may be responsible for a decrease of thoracic kyphosis [13, 14]. However, Hirsch et al. have shown that sublaminar bands induced a 55% vertebral derotation [8]. Even if derotation occurs with this technique, the main reduction principle is the posteromedial translation, allowing a significant correction in the sagittal plane. Hybrid constructs using sublaminar bands thus appear as a better technique for the restoration of sagittal alignment.

### 4.3. Changes on Adjacent Curvatures

In this series, the restoration of a normal thoracic kyphosis has resulted in changes on adjacent sagittal curvatures. There was no change in lumbar lordosis on immediate postoperative measurements but a significant increase was observed after the first 6 months. This change was stable at the final follow-up. These findings are consistent with those reported by Blondel et al. [15]. It demonstrates that lumbar lordosis is a parameter that secondarily adjusts to the new sagittal balance induced by the restoration of the thoracic kyphosis. Moreover, lumbar lordosis was significantly better in accordance with the pelvic incidence at 2-year follow-up, which confirms the influence of adjacent curvatures correction on global sagittal alignment. Changes have also been observed on pelvic parameters. The increase of pelvic version postoperatively reflects an adaptation of the pelvis to the new sagittal balance. These changes are transitory and at 2-year follow-up, pelvic version was the same as preoperative measurements. Moreover, a significant decrease in SVA has been observed. However, these changes do not seem to be clinically relevant because these young patients have excellent abilities to maintain overall sagittal alignment despite potential abnormal sagittal curvatures.

Concerning the cervical spine, there was a significant increase of the cervical lordosis from the immediate postoperative measurement to the final follow-up. Thoracic kyphosis restoration thus permitted changing from a 5° preoperative cervical kyphosis to a 9° cervical lordosis at 2-year follow-up, as described by Ilharreborde et al. [16]. In recent literature, it seems that cervical lordosis restoration has a major impact. Protopsaltis et al. have shown that this parameter was directly correlated to the scores of quality of life in adults [17]. A low value of thoracic kyphosis being at the origin of a kyphotic decompensation of the cervical spine is a major element to take into account when correcting this kind of spinal deformity [18].

Recent literature has shown the importance of sagittal alignment restoration when correcting scoliotic deformities [19–21]. More and more authors agree that the consideration of the sagittal plane in the surgical management of scoliosis is as important as the correction of the frontal plane [21, 22]. With the restitution of thoracic kyphosis, sublaminar bands allow improving adjacent curvatures of the spine and keeping a satisfying sagittal alignment.

## 5. Conclusion

The best construct to use in the management of patients with adolescent idiopathic scoliosis remains controversial. Hybrid construct with proximal hooks, distal screws, and sublaminar bands in the concavity is a frequently used strategy. Correction by posteromedial translation maneuvers by the tensioning of the sublaminar bands is efficient for the correction of both fontal and sagittal plane. Moreover, the restoration of normal thoracic kyphosis is followed by an adaptation of the adjacent curvatures with improved cervical lordosis and lumbar lordosis.

## Figures and Tables

**Table 1:** Radiographic parameters.

	Preoperative		Postoperative		6-month follow-up		2-year follow-up	
	Mean	SD	Mean	SD	Mean	SD	Mean	SD
Cobb angle	59.3	14.9	17.9	6.5	19.9	8	20.1	7
		**p** < 0.001		*p* = 0.003		*p* = 0.93		
Cervical lordosis	−5.6	11.5	3.5	9.5	4.3	12.3	9.4	10.5
		**p** = 0.001		*p* = 0.89		**p** = 0.02		
Thoracic kyphosis	10.5	7.8	24.1	7.2	26.6	8.1	27,0	8.6
		**p** < 0.001		*p* = 0.05		*p* = 0.2		
Lumbar lordosis	39.7	11.1	41.3	10.5	43.6	12.9	45.6	9.2
		*p* = 0.27		**p** = 0.002		*p* = 0.15		
